# A Case Report on QTc Prolongation: Understanding the Medication Risks and Electrolyte Imbalance

**DOI:** 10.7759/cureus.21421

**Published:** 2022-01-19

**Authors:** Shikha Jha

**Affiliations:** 1 Internal Medicine, Saint Peter's University Hospital, New Brunswick, USA

**Keywords:** qt prolongation, dizziness, hypokalemia, side effects of amiodarone, lonq qt syndrome, long qt

## Abstract

Prolonged QTc interval is one of the critical risk factors for sudden cardiac death. We all know that sudden cardiac death is often caused by acute onset ventricular arrhythmia, and QTc prolongation is one of the potential risk factors. It can be congenital or acquired. The acquired ones are commonly witnessed in day-to-day clinical practice. Several classes of mediations are well known to cause these conditions. Among many antiarrhythmic agents, especially amiodarone, is a critical drug to be monitored, as it strongly potentiates QTc prolongation. Especially in combination with metabolic abnormalities, this abnormality can occur rapidly with notable clinical presentation. This case report elicits an interesting clinical scenario in which a 79-year-old pleasant lady with multiple comorbidities presents with a syncopal episode. Missing the cardiologist’s appointment for dose adjustments of her medication, amiodarone was noteworthy. Also, an acute electrolyte imbalance from the possibly recent use of diuretics aggravated the clinical situation. On presentation, the electrocardiogram showed a remarkably prolonged QTc, which was way more compared to the prior ones available. Discontinuation of amiodarone and repletion of the electrolytes brought down the QTc interval to almost a normal range and no syncopal episode within two days. Hence, understanding the medications’ potential risks and having a close watch on the possible side effects is key to avoiding dreadful complications of arrhythmia and sudden cardiac death from the same. This case report cumulatively covers this essential medical knowledge and practical, vital points.

## Introduction

The QTc prolongation seen on an electrocardiogram (EKG) is caused by the alteration in the repolarization of the myocardium. This can lead to a constellation of either congenital or acquired syndrome or both. The patients with this abnormality primarily present with syncopal episodes, palpitations, dizziness, seizure episode, or in severe cases, sudden death. The syndrome is predisposed to the increased chances of dreadful arrhythmia, torsades de pointes, a polymorphic ventricular tachycardia. QTc prolongation is an independent risk factor for sudden cardiac death [1]. While congenital is due to hereditary abnormalities related to repolarization, the acquired form is most commonly due to drug treatment and metabolic abnormalities. The drug therapies leading to acquired QTc prolongation are antiarrhythmic, antianginal, anticholinergic, antimicrobials, antineoplastic, androgen deprivation therapy, analgesic, anesthetic, sedative, diuretics, antiemetics, prokinetics agents, and many more [2]. Amiodarone, a class III antiarrhythmic, is well known for remarkable QTc prolongation. Typically, when hypokalemia is present concomitantly with amiodarone use, it amplifies the risk of torsades de pointes and sudden cardiac death [3]. The incidence of QTc prolongation without torsades de pointes is much higher than torsades de pointes itself.

## Case presentation

**Figure 1:**
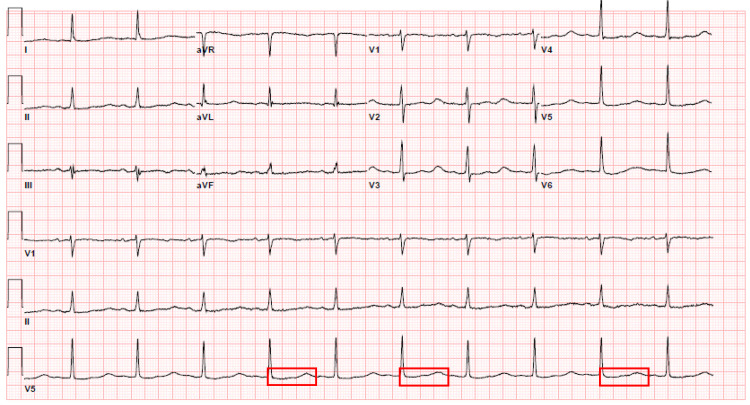
Electrocardiogram on admission - Corrected QT interval 692 milliseconds, demarcated by multiple red boxes.

The patient is a 79-year-old female who presented to the hospital with a chief complaint of the syncopal episode. She stated that she “almost fainted” the night before while at rest. Her family members had witnessed the episode. They told her that she regained her consciousness in less than a minute. No associated symptoms of chest pain, palpitations, shortness of breath, body weakness, nausea, vomiting. Her medical history was significant for paroxysmal atrial fibrillation, hypertension, hyperlipidemia, lower extremity swelling, pulmonary hypertension, and obstructive sleep apnea. Home medications included amiodarone, atorvastatin, amlodipine, Lasix and Xarelto. One of her medications, metoprolol, was discontinued a couple of months ago, by her cardiologist, due to persistent bradycardia. She stated that she was supposed to see her cardiologist for dose adjustments of amiodarone last month. However, she planned on postponing the visit. She has been on oral amiodarone 20 mg once daily for the past few months. Physical exam elicited regular S1 and S2, with no murmurs, normal neurological exam, orthostatic negative, and no edema noted. The Electrocardiogram (EKG) on admission showed normal sinus rhythm with 62 beats per minute ventricular rate. The corrected QTc interval was 692 milliseconds [Figure 1].

The proper investigations, including lab investigations and images. Out of all the investigations, a low potassium level of 3.1 millimole/liter was noteworthy (Table 1).

**Table 1:** Labs and Imaging. The potassium level is 3.1 millimoles/liter (low).

PERTINENT INVESTIGATIONS	RESULTS
Cardiac troponin with a trend	<0.03 nanogram/mililiter
Complete blood count with differential	Values within normal range
Coagulation profile	Values within normal range
Serum Potassium level	3.1 millimole/liter
Serum Sodium level	Values within normal range
Serum Magnesium level	Values within normal range
Serum Creatinine level	Values within normal range
Corrected Calcium level	Values within normal range
Chest X ray	No evidence of acute cardiopulmonary abnormality.
Cardiac Echocardiogram	Left ventricular ejection fraction of 60%, concentric left ventricular hypertrophy with right ventricular systolic pressure of 39 mm Hg.

The patient was put on continuous cardiac monitoring because of prolonged QTc interval and a syncopal episode. The plan was to eradicate the triggering agents and treat the underlying etiologies. The patient’s home medication, amiodarone, was discontinued. Given acute hypokalemia, the diuretic was held, as it would further worsen the drop in the potassium level via renal excretion. The potassium levels were brought back within the normal range (4.2 millimole/liter with adequate oral repletion, using potassium chloride tablets, and providing a potassium-rich diet. 

**Figure 2:**
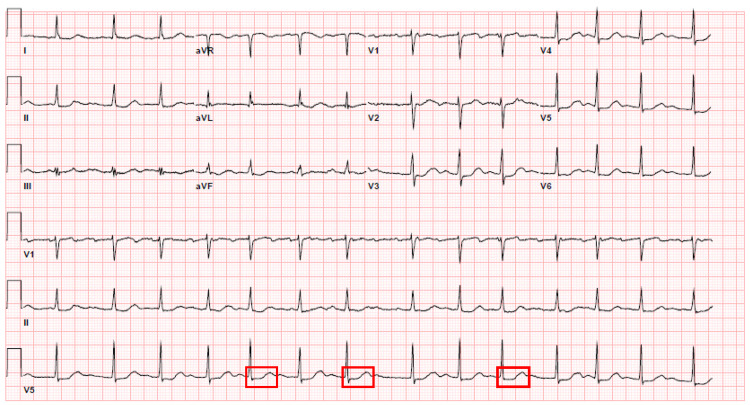
Electrocardiogram 2 days later - Corrected QTc interval of 501 milliseconds, demarcated by multiple red boxes

After taking these prompt steps of management, a follow-up electrocardiogram was done. The electrocardiogram showed a corrected QTc interval of 501 milliseconds (Figure 2). 

This electrocardiogram showed a drastic decrement of prolongation by 191 milliseconds compared to the initial one, moving to restore the interval to the normal range. The patient did have a known history of paroxysmal atrial fibrillation from before [Figure 2]. The subsequent electrocardiogram showed a normal sinus rhythm with a QTc interval of 484 milliseconds. She had no active cardiorespiratory symptoms during these repeat electrocardiograms. The patient has no further syncopal episodes during her hospital stay. The patient was well explained about the entire medical event as described above. She was counseled about the medication-related side effects with the teach-back mechanism. She was told that she would need a timely and prompt follow-up with the cardiologist for revision of her medications, repeat electrocardiogram and close electrolyte monitoring.

## Discussion

The QT interval in an electrocardiogram indicates the time frame of the ventricular action potential, which corresponds to the depolarization and repolarization of the ventricles [4]. The QT interval varies with the heart rate; hence it is crucial to correct the QT interval for a particular heart rate. This is called corrected QT or QTc interval. There are several formulas available to calculate the QTc interval. The Bazett formula, one of the most common ones, QTc = QT / √RR, has been used for this patient. The QTc value is abnormal if it is more than 440 milliseconds in males and more than 460 milliseconds in females. Syncope is the most common complaint of the patient with QTc prolongation. Our patient, in this case, presented with a syncopal episode, which is likely from the prolonged QTc interval consequences [5]. In such a clinical situation, it is equally important to rule out other potential causes of syncope via good history taking and physical exam. Syncopal episodes can be caused by several etiologies that are classified as neurally medicated, cardiovascular, and orthostatic causes. The physical exam of our patient’s case did not have any findings that would indicate an orthostatic or reflex-mediated cause.

The next step of doing an electrocardiogram helped us detect the prolonged QTc interval, 692 milliseconds. It is the most likely cause of this patient’s presenting complaint. Once prolonged QTc is detected, the best next step is to identify the underlying triggers or causes. The acquired cause of QTc prolongation is more common than the congenital cause. Medication side effects and electrolyte disturbances are the most common causes of acquired QTc prolongation. Medications that lead to prolonged QTc are antiarrhythmics, antimicrobials, psychotropics, antineoplastic antihistamines, antiemetics [6]. Metabolic disturbances that led to QTc prolongation are hypokalemia, hypomagnesemia, and hypocalcemia. In this case, our patient has two prominent risk factors, amiodarone uses and evidence of hypokalemia [7]. The antiarrhythmic medication, amiodarone, has a pertinent effect of potassium channel blocking during repolarization. This leads to the prolongation of the QTc interval. Additionally, the loop diuretics led to QTc prolongation via potassium diuresis. The patient in the above case has all these risk factors present, identifying the etiologies to her presenting condition.

The most dreadful complication of QT prolongation is torsades de pointes, a pattern of polymorphic ventricular tachycardia [8]. It is a lethal form of arrhythmia. The premature contraction of ventricles is typical in this scenario. Consequently, this arrhythmia may degenerate into ventricular fibrillation. If not intervened promptly, it may lead to death. Hence, the management should focus on acute events and long-term care. The initial step should be to place the patient under continuous telemetry monitoring. Hospitalization is a must for patients with active symptoms and remarkable prolongation of more than 500 milliseconds. Identifying the etiology, including at-risk medications, and withholding them is the next best step. Also, close monitoring of electrolytes with adequate repletion and recheck is vital to avoid electrolyte imbalances. If the patient develops complications of bradyarrhythmia or pause-dependent torsades de pointes, a permanent pacemaker may be required.

For long-term care, patients should be well educated about the potential drugs that could lead to QTc prolongation if they are on any of them. Also, they should be taught to identify the likely symptoms that they develop from QTc prolongation and report promptly. Clear and thorough communication should be with the patient regarding the benefits of using QTc prolonging medication. It should be based on analyzing the risk-benefit ratio and individual cases. The aim should always consider three risk factors, patient-related, medication-related, and interaction with other risk factors related.

## Conclusions

Diagnosis and management of QTc prolongation should be made promptly. It can be challenging and life-threatening if not reported and intervened on time. Medications with dreadful side effects require a close watch, mainly if they exist together with several other risk factors. Medications such as amiodarone prolong QTc interval. Another medication, furosemide, provokes hypokalemia via potassium diuresis, prolonging QTc. Both these medications and their subsequent side effects, including electrolyte imbalance, have been covered in this case report. Educating the patients about the medication-related risks and their potential side effects is as essential as prescribing them. Understanding alarming symptoms from those side effects and taking prompt action is crucial. It is a cumulative approach, from both physician and patient, to work together at preventive and corrective steps to avoid QTc prolongation-related manifestations as well as complications.
